# A multicentre survey of Australian intensivists on optimal use of midazolam for sedation

**DOI:** 10.1016/j.ccrj.2026.100184

**Published:** 2026-05-12

**Authors:** Jarrod Rawson, Maurice LeGuen, Ashwin Subramanian, Andrew Udy

**Affiliations:** a Department of Intensive Care and Hyperbaric Medicine, Alfred Health, Melbourne, Victoria, Australia; b Department of Intensive Care, Monash Health, Dandenong, Victoria, Australia; c Australian and New Zealand Intensive Care Research Centre, School of Public Health and Preventive Medicine, Monash University, Melbourne, Victoria, Australia; d Department of Intensive Care, Epworth Hospital, Richmond, VIC, Australia; e Peninsula Clinical School, Monash University, Frankston, VIC, Australia

**Keywords:** Midazolam, Sedation, Intensive care, Benzodiazepines, Analgosedation, Clinical decision-making

## Abstract

**Background:**

Midazolam is widely used for sedation in intensive care units (ICUs). Emerging evidence links benzodiazepines with prolonged mechanical ventilation, increased delirium, and longer ICU stays. Clear guidance defining the role of midazolam in modern practice remains limited, and its use is largely clinician dependent.

**Objective:**

The aim of this study was to describe contemporary Australian intensivist opinions regarding indications, patient and disease modifiers, titration, and weaning strategies for midazolam infusions.

**Participants:**

Australian ICU specialists and fellows were included.

**Design:**

We conducted a national cross-sectional self-administered survey. A 73-item questionnaire using case vignettes and Likert-scale responses assessed first-line and second-line indications, modifiers influencing use, and cessation strategies for midazolam infusions. Strong Agreement/Disagreement was defined *a priori* as greater than 80% of respondents indicating a score of 4 or 5 (Strongly agree; Agree) or a score of 1 or 2 (Strongly disagree; Disagree).

**Results:**

Sixty-one clinicians responded. No first-line indications for midazolam use reached strong agreement. Respondents strongly disagreed with midazolam use as first-line sedation in agitation (90.1%, n = 55/61), acute respiratory distress syndrome (81.6% n = 49/60), positional restrictions (83.6%, n = 51/61), and out-of-hospital cardiac arrest (91.6%, n = 55/60). Strong agreement supported discontinuing midazolam when commenced prior to ICU admission (82%, n = 50/61). Strong agreement supported midazolam use as second-line sedation in traumatic brain injury (90%, n = 54/60) and status epilepticus (98.3%). No agreement was achieved for patient or disease modifiers, demonstrating substantial practice variability. For weaning, there was strong agreement that midazolam should be the first sedative ceased when used with other agents (90%, n = 54/60) and that tapering was unnecessary with infusions shorter than 48 h (80%, n = 47/59).

**Conclusions:**

Australian ICU practitioners report being selective with the use of midazolam and convey it is primarily reserved for use as a second-line agent in traumatic brain injury and status epilepticus. Persistent heterogeneity highlights the need for clearer guidance on selective use and weaning in modern ICU practice.

## 1 Introduction

Midazolam is a potent benzodiazepine, commonly used for sedation in Australian intensive care units (ICUs) due to its hypnotic, anxiolytic, amnestic, anticonvulsant, and pharmaceutic properties.[1], [2], [3] It is cost-effective and is useful in providing deep sedation with only a modest reduction in systemic vascular resistance.^2^ Despite being a short-acting benzodiazepine when dosed intermittently, a prolonged recovery time with continuous infusions is noted due to the longer half-life during the redistribution and elimination phases.^2^^,^^4^

Despite ongoing use over decades, recent evidence, especially from the MIDEX-PRODEX (Dexmedetomidine vs Midazolam or Propofol for Sedation During Prolonged Mechanical Ventilation) and SEDCOM (Safety and Efficacy of Dexmedetomidine compared with Midazolam) trials, indicated that midazolam was associated with adverse outcomes compared to newer and shorter-acting agents such as propofol and dexmedetomidine.^5^^,^^6^ These included prolonged mechanical ventilation, longer ICU stays,^7^^,^^8^ and ICU-related delirium.^9^^,^^10^ While not consistent across studies, Shi et al. and Sun et al. suggested midazolam was associated higher ICU mortality, though this finding was not validated in systematic review and meta-analysis.[11], [12], [13], [14]

As such, the use of midazolam is based on patient indication, sedation goals, and its pharmacology compared to other sedatives.^13^ It is commonly used for early sedation in traumatic brain injury (TBI) and seizures but its use in cases like drug-related agitation or specific areas such as trauma and burns is less well established. Prolonged infusions or use in renal or liver dysfunction can cause prolonged sedation; therefore, careful consideration is required when weaning.^13^ Contraindications must be considered due to the risks of accumulation and extended effects.^13^

Currently, the use of midazolam in modern ICU sedation protocols remains largely at the discretion of the treating clinician as formal guidelines are lacking. The Society for Critical Care Medicine Pain, Agitation/Sedation, Delirium, Immobility and Sleep (PADIS) Guidelines recommend propofol or dexmedetomidine over benzodiazepines, including midazolam, for sedation in ICU patients, owing to the higher risk of delirium and prolonged sedative effects associated with benzodiazepine use.^15^ A conditional recommendation is made to limit use to 48 h when able.^15^ Therefore, midazolam is not recommended as a first-line choice in routine ICU patients, where shorter and lighter periods of sedation are intended.[16], [17]

International guidelines provide limited advice regarding the use of benzodiazepines in certain subspecialty populations. One notable exception is the recommendation to avoid midazolam in cardiothoracic patients as these individuals typically require brief intubation periods.^15^ Neurosurgical guidelines, like those mentioned by the Brain Trauma Foundation and Neurocritical Care Society, prefer light-sedation targets which are more achievable with nonbenzodiazepine agents.^18^^,^^19^ However, in clinical practice, there are many subspecialty populations that require nuanced sedation regimes that are not covered by existing critical care sedation guidelines.

Therefore, we aimed to further define the contemporary role of midazolam in ICU sedation algorithms through a survey of ICU specialists and fellows across Australia. We sought to describe clinician-reported attitudes towards the use of midazolam in the ICU, including views around (i) indications; (ii) patient and disease modifiers that act as contraindications; and (iii) titration and weaning methods. Based on these findings, we assessed whether any of these aspects reflected agreement regarding the optimal usage of midazolam in the ICU.

## 2 Methods

### 2.1 Study design, setting, sample

We conducted a national cross-sectional multicentre self-administered survey sent to intensive care doctors of Australian adult ICUs. We report our findings according to the consensus-based checklist for reporting survey studies.^20^ Low-risk ethics approval was provided by the Alfred Health Hospital Research Ethics Committee (Project 530/24).

Specialists and fellows working in Australian ICUs were invited to participate, indicating high-level experience in sedation practices. Our sample frame was generated by using contact details for unit heads and research leads of ICUs across all Australian states as well as existing professional contacts. The survey was also promoted on the College of Intensive Care Medicine website. An arbitrary sample size of 50 participants was set as a cut-off for analysis to ensure adequate representation across participating Australian centres, providing sufficient response volume to meaningfully interpret practice variability while remaining feasible for a multicentre survey design.

### 2.2 Survey development

Our investigator team (one ICU fellow and three specialists) generated potential survey items considering midazolam use within specific patient groups and disease states regularly encountered within ICUs. We linked content with case vignettes to provide clinical context to each proposition and to evaluate subspecialty ICU populations and specific patient and disease modifiers (Appendix). The survey was developed through literature review, refined for face and content validity with expert input, and piloted with two fellows to ensure clarity, relevance, and ease of completion.

### 2.3 Survey administration

A survey link and participant information brochure was emailed to Australian ICUs through contacts at each location. Participants were encouraged to forward the link to professional networks of ICU medical practitioners. Reminders were sent to nonresponders at 1 and 3 months post the initial invite. An initial invite was sent on 23 April 2025, and the survey was closed on 26 October 2025. No follow-up surveys were planned. Due to this indirect distribution method and secondary forwarding, it was not possible to accurately determine the denominator of recipients and response rate. We estimate 200 or more clinicians received the invite.

### 2.4 Survey content

A 73-item survey was developed that explored perceptions about (i) specific indications (first line and second line); (ii) patient and disease modifiers; and (iii) tapering and cessation techniques for prolonged midazolam infusion (defined as >12 h of use). Case vignettes applied to only the first 48 h of ICU admission. Subsequent questions related to continued midazolam infusion for up to a week.

### 2.5 Data collection and management

Data were collected anonymously, and participation was entirely voluntary. Institution names were not recorded. No financial incentives were offered. Completion of the survey was considered implied consent, as outlined on the survey landing page. Answers to propositions were in the form of a Likert scale of agreement/frequency of use in that setting (Strongly disagree: 0–15%; disagree: 16–30%; neutral: 31–70%; Agree: 71–85%; Strongly agree: 86–100%).

### 2.6 Definitions

First-line sedation was defined as the initial choice of sedation that would be used as monotherapy (+/- opioid) if sedation targets are achieved. Second-line sedation was defined as choice of sedation that would be used in addition to another agent (usually propofol).

Predetermined cut-offs for strong agreement/disagreement were decided *a priori* as greater than 80% of respondents indicating a score of 4 or 5 (Strongly agree; Agree) or a score of 1 or 2 (Strongly disagree; Disagree). Weak agreement/disagreement was defined retrospectively as 70–80% selecting a score of 4 or 5 (Strongly agree; Agree) or a score of 1 and 2 (Strongly disagree; Disagree).^21^

### 2.7 Statistical analysis

Data analysis was performed using primarily descriptive methods. Survey responses were reported as percentages and proportions of valid responses as well as median score response. Results were thematically displayed in radar plots and bar charts of strong and weak agreement. All analyses were conducted as complete case analyses, and any missing data were handed by a case-wise deletion in the individual analyses.

## 3 Results

### 3.1 Demographics

A total of 61 ICU clinicians responded to the survey, all of which were analysed. Fifty-nine of these completed the survey in full. Most survey respondents had over 5 years of ICU specialist experience (68.8%) and were from either Victoria (55.7%) or New South Wales (26.2%). Most respondents worked in a tertiary/quaternary ICU (83.6%) and looked after one or more ICU subspecialty patient groups (Table 1).

**Table 1:** Demographics of survey respondents.

	Number (n = 61)	Percentage
**Experience**		
- Fellow	6	9.8
- Consultant 1–2 years	6	9.8
- Consultant 2–5 years	7	11.4
- Consultant >5 years	42	68.8
**Location**		
- Australian Capital Territory	1	1.6
- New South Wales	16	26.2
- Northern Territory	1	1.6
- Queensland	0	0
- South Australia	5	8.2
- Tasmania	0	0
- Victoria	34	55.7
- Western Australia	0	0
**ICU category**		
Rural/regional	4	6.6
Metropolitan	6	9.8
Tertiary/quaternary	51	83.6
Private	0	0
**Subspecialties cared for in the ICU**		
Burns	31	50.1
Mechanical circulatory support	46	75.4
Neurosurgery	54	88.5
Trauma	50	81.2

ICU: intensive care unit.

### 3.2 Indications

**Fig. 1:**
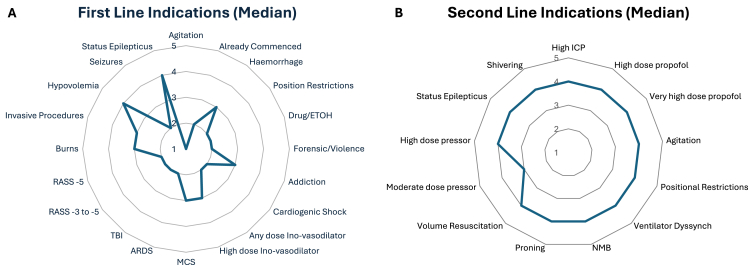
Median results (Score 1–5) for questions regarding: First-line (A) and second-line indications (B) for midazolam. Abbreviations: ARDS: adult respiratory distress syndrome; ETOH: alcohol; ICP: intracranial pressure; MCS: mechanical cardiac support; NMB: neuromuscular blockade; RASS: Richmond Agitation and Sedation Scale; TBI: traumatic brain injury.

**Fig. 2:**
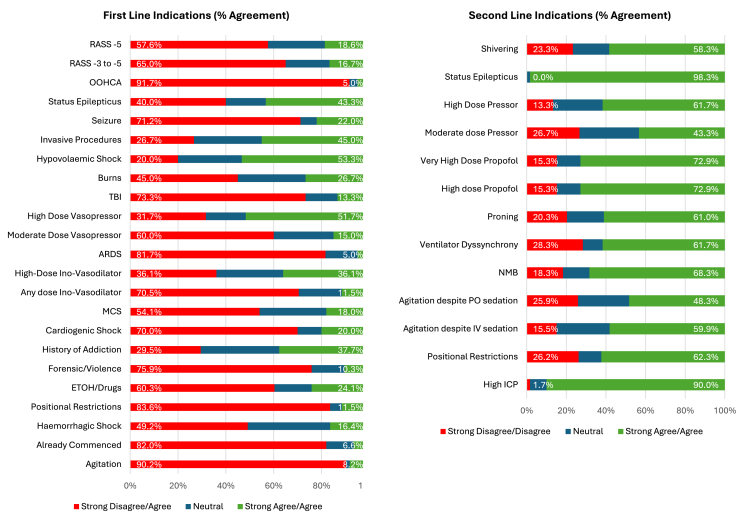
First- and second-line indications for midazolam use (combined percentages of strongly disagree/disagree and strongly agree/agree shown). Abbreviations: ARDS: acute respiratory distress syndrome; EtOH: alcohol; ICP: intracranial pressure; IV: intravenous; MCS: mechanical circulatory support; NA: noradrenaline; NMB: neuromuscular blockade; OOHCA: out-of-hospital cardiac arrest; RASS: Richmond agitation and sedation score; TBI: traumatic brain injury.

Median ratings for each indication are illustrated in Fig. 1, Fig. 2 and Table 2. Survey respondents strongly disagreed with the use of midazolam as a first-line agent in intubated patients with agitation (90.1% disagreement, n = 55/61), out-of-hospital cardiac arrest (91.6% disagreement, n = 55/60), positional restrictions (83.6% disagreement, n = 51/61), or for acute respiratory distress syndrome (ARDS; 81.6% disagreement, n = 49/60). There was also strong disagreement that midazolam should be continued in the ICU if it has already been commenced elsewhere (82% disagreement, n = 50/61). No other indications reached strong agreement or disagreement for first-line use. In contrast, strong agreement supported midazolam as a second-line agent in TBI (90% agreement, n = 54/60) and status epilepticus (98.3% agreement, n = 59/60).

**Table 2:** Summary of strong and weak agreement results.

	Strong Agreement/Disagreement	Weak Agreement/Disagreement
First-line indications	- Agitation (90.1% disagreement) - Out-of-hospital cardiac arrest (91.6% disagreement) - Positional restrictions (83.6% disagreement) - Acute respiratory distress syndrome (81.6% disagreement) - Discontinue if has been commenced elsewhere (82% agreement)	- Seizures (71.2% disagreement) - TBI (73.3% disagreement) - Patients requiring inovasodilators (70.5% disagreement) - Cardiogenic shock (70% disagreement) - Forensic or violence history (75.8%) - History of drug addiction (70.5% agreement) - Sedation targets are not achieved despite doses of propofol >200–250 mg/h (72.9% agreement)
Second-line indications	- Traumatic brain injury (TBI) (90% agreement) - Status epilepticus (98.3% agreement)	-
Patient and disease modifiers	-	-
Titration and weaning strategies	- First agent weaned (90% agreement) - No need to taper the dose following a short-term infusion (<48 h) (80% disagreement)	- Gradually tapered when used for greater than 48 h (70% agreement) - Development of tolerance is a concern in prolonged infusion (78% agreement)

Respondents weakly disagreed with the use of midazolam first-line in seizures (71.2%, n = 42/59), TBI (73.3%, n = 44/60), cardiogenic shock (70%, n = 43/60), patients requiring inovasodilators (70.5%, n = 45/50), and those with forensic or violent histories (75.8%, n = 45/61). Weak agreement supported its use in patients with substance use disorders (70.5%, n = 43/61) or when sedation targets were not achieved despite high-dose propofol (>200–250 mg/h; 72.9%, n = 43/59).

### 3.3 Patient and disease modifiers

**Fig. 3:**
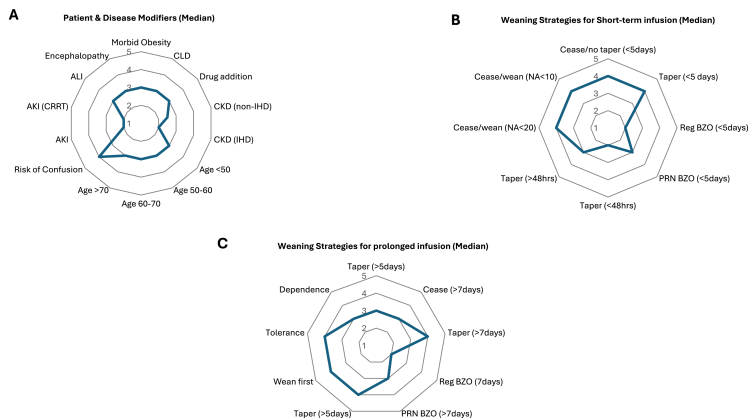
Median results (score: 1–5) for questions regarding patient and disease modifiers (A), weaning strategies for short-term (B) and prolonged (C) infusions. Abbreviations: AKI: acute kidney injury; ALI: acute liver injury; BZO: benzodiazepine; CKD: chronic kidney disease; CLD: chronic liver disease; CRRT: continuous renal replacement therapy; NA: noradrenaline; PRN: as required; Reg: regular.

**Fig. 4:**
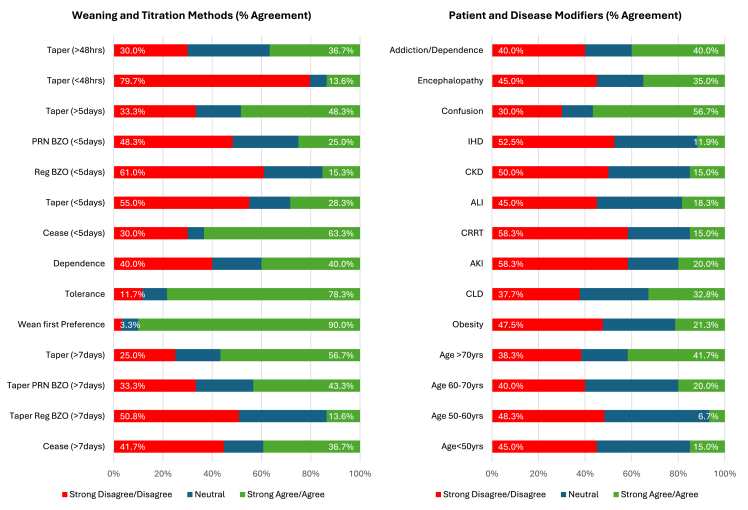
Patient and disease modifiers and weaning and titration strategies for midazolam. Abbreviations: ALI: acute liver injury; CKD: chronic kidney disease; CRRT: continuous renal replacement therapy; IHD: intermittent haemodialysis; PRN BZO: benzodiazepine as required; reg: regular.

Median ratings for patient- and disease-specific modifiers are presented in Fig. 3 (panel A), Fig. 4, and Table 2. No strong or weak agreement was reached regarding patient- and disease-specific modifiers, especially relative haemodynamic stability, shock, or vasopressor requirements.

### 3.4 Titration and weaning strategies

Median ratings for titration and weaning strategies are presented in Fig. 3 (panels B and C), Fig. 4, and Table 2. Respondents strongly agreed that midazolam should be the first sedative weaned when used alongside other agents (90% agreement, n = 54/60). Similarly, there was strong disagreement with the suggestion tapering was necessary with short-term infusions (<48 h) (80% disagreement; n = 47/59). Weak agreement supported gradual tapering after prolonged use (>48 h) (70% agreement, n = 42/60) and recognised tolerance as a concern with very prolonged infusions (78% agreement, n = 47/60). No agreement was reached for other weaning strategies.

## 4 Discussion

### 4.1 Executive summary

In this multicentre survey of Australian ICU clinicians, respondents indicated a more selective approach to the use of midazolam, strongly disagreeing with the concept of using midazolam first line in mechanically ventilated patients with agitation, cardiac arrest, position restrictions, and ARDS. However, midazolam continued to have a clearly supported second-line role in selected neurological indications, notably in TBI and status epilepticus. Clinicians did not reach agreement on specific patient or disease modifiers that should guide or restrict its use, reflecting heterogeneity in practice and contextual decision-making. Strong agreement was observed regarding weaning strategies, with midazolam preferentially discontinued first among sedatives, with no taper required following short-term use, while prolonged infusions warrant gradual tapering due to concerns for tolerance. Overall, these results suggest that Australian ICU clinicians may no longer perceive midazolam as a broad-spectrum sedative and instead primarily reserve its use for more specific neurological emergencies.

### 4.2 Comparison with published literature

To the best of our knowledge, this is the first study to describe the contemporary attitudes of ICU practitioners to the use of midazolam in the context of widely available nonbenzodiazepine alternatives. While strong agreement was achieved in a few areas, several additional propositions reached “weak” agreement, suggesting that midazolam may still be used across a range of indications. This pattern likely reflects substantial practice heterogeneity among Australian ICUs, driven by factors such as gaps or ambiguity in the evidence base, lack of clear operational guidance; overlap with other sedatives agents, the need for individualised sedation decisions; variability in ICU clinicians’ exposure; and training- and unit-specific sedation culture.

Our findings align closely with international guideline recommendations, including the Society for Critical Care Medicine PADIS guidelines, which favour nonbenzodiazepine sedatives due to associations between benzodiazepines and delirium, prolonged ventilation, and delayed awakening.^15^ This survey identified two indications with strong agreement for second-line use of midazolam, with several others achieving only weak agreement. Benzodiazepines are preferred for their sedative and anticonvulsant effects in TBI and status epilepticus, but respondents agreed midazolam should not be used first line, potentially to allow opportunities for neurologic assessment. The absence of agreement for first-line use of deep sedation in out-of-hospital cardiac arrest, positional restrictions, and ARDS suggests a shift away from older deep-sedation protocols and reinforces the principle of early sedation reassessment at ICU admission.

Despite biological plausibility for midazolam use in haemodynamic instability, the survey did not identify clear thresholds for its initiation or cessation in shocked patients, with limited agreement across shock types and inovasodilator/vasopressor doses, likely reflecting both practice variability and the heterogeneous nature of shock.^2^ In practice, sedative choice in this setting involves a trade-off between short-term haemodynamic stability and longer-term risks such as delirium and delayed extubation, further influenced by underlying pathophysiology, concurrent therapies (e.g., vasopressin), disease trajectory, and required depth of sedation. These findings highlight the lack of high-quality evidence to guide sedative choice in haemodynamically unstable patients and support the need for further work to better define midazolam-prescribing priorities in this setting.

In contrast, greater agreement was observed for cessation and titration practices: midazolam was preferred as the first sedative to be weaned when used concurrently with other agents, tapering was considered unnecessary after short-term infusions (<48 h), and continuation of midazolam commenced prior to ICU admission was discouraged without a clear indication. Overall, these findings suggest that Australian ICU doctors generally limit midazolam use in line with guideline preferences for nonbenzodiazepine sedation, while substantial variability persists across several clinical indications.

Notably, no patient- or disease-specific modifiers reached either strong or weak agreement as relative contraindications to midazolam use, highlighting substantial practice heterogeneity in Australian intensivists. This contrasts with guideline cautions, particularly the PADIS guidelines, regarding increased delirium risk and prolonged sedation in high-risk groups such as older adults and patients with cognitive, hepatic, or renal impairment. The findings suggest that ICU practitioners may still consider midazolam in these populations, although it remains uncertain whether dosing or duration is systematically adjusted to mitigate risk. Further research is needed to better define which patient subgroups benefit from or are potentially compromised by midazolam exposure.

### 4.3 Strengths and limitations

A key strength of this study is the breadth of survey questions, which enabled a comprehensive examination of multiple dimensions of midazolam use. The inclusion of case vignettes to provide clinical contexts potentially enhanced the realism of responses and improved their alignment with actual clinical practice.^22^ While the survey included only 61 respondents, this sample size was comparable to other Australian surveys of intensive care specialists, and the high completion rate (98%) supports data completeness and engagement.[23], [24], [25] Importantly, the opinions expressed reflect the perspectives of experienced intensivists from across Australia (predominantly Victoria) with significant experience in the care of subspecialty ICU patients.

This study has several limitations. Participation was voluntary, introducing potential response bias as clinicians with greater interest in sedation practices may have been more likely to respond. Due to indirect distribution and secondary forwarding, the true response rate and denominator of recipients could not be determined. Geographic representation was uneven, with predominance of respondents from New South Wales and Victoria, as well as tertiary hospitals, limiting the ability to infer national prescribing patterns. To preserve anonymity, workplace identifiers were not collected, preventing assessment of clustering effects or the number of respondents per ICU; responses from clinicians within the same unit may therefore be correlated due to shared policies or culture. The modest sample size further limits representativeness, although this is comparable to other survey-based studies within the relatively small Australian ICU specialist workforce.^26^^,^^27^ As a self-reported survey, results reflect clinician preferences rather than observed prescribing behaviour and may be influenced by recall and social desirability bias, particularly given guideline recommendations favouring nonbenzodiazepine sedation. Predefined survey items and case vignettes may also incompletely capture the complexity of bedside decision-making. Finally, the study did not assess dosing strategies, duration of exposure, or patient outcomes, limiting conclusions regarding the clinical effectiveness or harm associated with midazolam use.

## 5 Conclusion

This survey suggests Australian ICU clinicians now reserve midazolam for specific neurological conditions like TBI and status epilepticus rather than as a first-line sedative for ventilated patients. Significant variation remains in practice, especially regarding cessation and weaning and contraindications. These results underscore the need for more research on midazolam’s selective use, safe parameters, and optimal patient groups.

## CRediT authorship contribution statement

**Jarrod Rawson:** Conceptualisation, Methodology, Formal Analysis, Writing- Original Draft. **Maurice LeGuen:** Conceptualisaton, Methodology, Investigation, Writing- Review and Editing, Supervision. **Ashwin Subramanian:** Methodology, Visualisation, Supervision, Writing- Review and Editing. **Andrew Udy:** Supervision, Writing- Review and Editing. The authors received no financial support for the research, authorship and publication of this article.

## Conflict of interest

The authors declare the following financial interests/personal relationships which may be considered as potential competing interests: AS and AU are Associate Editors / Editorial Board Members for Critical Care and Resuscitation Journal. Other authors do not have any conflicts of interest to declare.

If there are other authors, they declare that they have no known competing financial interests or personal relationships that could have appeared to influence the work reported in this paper.

## Figures and Tables

**Table:** 

	Likert scale	Frequency of use	Likelihood to use midazolam
1	Strongly disagree	0–15%	Very unlikely
2	Disagree	16–30%	Less likely
3	Neutral	31–70%	No change
4	Agree	71–85%	More likely
5	Strongly agree	86–100%	Very likely

**Table:** 

Question	Text	Yes	No
Domain 1: Consent and demographics			
1	***Do you consent for your opinion to be anonymously used as part of this Delphisurvey/quality improvement process?***		
2	***How many years have you been working as a senior clinician?***		
	*Fellow 1-2*		
	*Consultant 1-2*		
	*Consultant 2-5*		
	*Consultant 5*+		
3	***State/territory of practice:***		
	Victoria		
	New South Wales		
	Queensland		
	Northern Territory		
	Western Australia		
	South Australia		
	Australian Capital Territory		
	Tasmania		
4	***Level of ICU that most of your practice is in***		
	Secondary		
	Tertiary		
	Quaternary		
5	***In your daily practice, do you care for the following ICU subspecialty groups:***		
	Neurosurgical		
	Trauma		
	Burns		
	Mechanical cardiac support		

**Table:** 

	Case 1: Agitation and trauma (nontraumatic brain injury [TBI])
	*A 36-year-old male patient is admitted to your unit following a high-speed motor vehicle accident and was intubated in the emergency department (ED) for agitation. The initial CT brain is normal. Regarding sedation in this patient*
1.1	Midazolam as first-line sedation in patients presenting with agitation (i.e., pre-emptive/prophylactic)
1.2	I would continue midazolam if already commenced by another clinician (e.g., in the ED)
	*In the ICU, he is increasingly hypotensive. You are concerned for occult haemorrhage. Regarding sedation in this situation:*
1.3	Midazolam as first-line sedation in patients with uncontrolled haemorrhage
	*CT imaging confirms an unstable C-Spine fracture requiring a C-Spine collar to be fitted. Additionally, thoracic and lumbar spine are not yet cleared. You are concerned that without sedation, the patient will not be able to adhere to spinal precautions. Regarding sedation in this patient*
1.4	Midazolam as first-line sedation where strict position restrictions are required (i.e., the spine has not been cleared)
1.5	Midazolam as second-line sedation where strict position restrictions are required (i.e., the spine has not been cleared) and the initial choice of sedation is not adequate
	*Despite the initial propofol sedation, the patient remains highly agitated. This patient is found to have a history of heavy alcohol use and was intoxicated during the motor vehicle accident. You have been informed that he also has a forensic history and known to be violent. Regarding sedation for this patient*
1.6	Midazolam as first-line sedation if patient has history of drug or alcohol use
1.7	Midazolam as first-line pre-emptively/prophylactically in a patient with forensic/violence history
1.8	Midazolam as second-line sedation in patients who are intubated and agitated who do not respond to another intravenous sedative (e.g., propofol)
1.9	Midazolam as second-line sedation in patients who are intubated and agitated who do not respond to enteral sedation (e.g., quetiapine)
	*Rate your opinion whether your decision to use midazolam for this patient would change in the presence of one of the following:*
1.10	Morbid obesity (body mass index >35)
1.11	Chronic liver disease (Child–Pugh B or C)
1.12	With history of addiction to prescription drugs

**Table:** 

	Case 2: Cardiogenic shock
	*A 67-year-old male with ischaemic cardiomyopathy (ejection fraction: 25%) presents with acute chest pain, dyspnoea, and hypotension (blood pressure: 82/*50 mmHg*, heart rate: 110 bpm). He is diagnosed with cardiogenic shocksecondary to an acute myocardial infarction and undergoes emergent percutaneous coronary intervention (PCI) for left anterior descending Artery (LAD) occlusion. He returns to the ICU intubated and sedated, with blood pressure supported with milrinone 20 μg/min (0.5 μg/kg/min) and an Impella® (Abiomed). Regarding sedation in this patient*
2.1	Midazolam as first-line sedation in cardiogenic shock of any severity
2.2	Midazolam as first-line sedation when using an inovasodilator (e.g., milrinone)
2.3	Midazolam as first-line sedation when requiring mechanical cardiac support
2.4	Midazolam when requiring high dose of inovasodilator (e.g., milrinone >20 μg/min or >0.5 μg/kg/min) or second inotrope
	*Due to the severe shock state,this patient has ischaemic Liver Function Test (LFT) derangement, is oliguric and delirious. Rate your opinion of whether your decision to use midazolam in this patient would change in the setting of any of the following organ impairments:*
2.5	Acute kidney injury Glomerular Filtration Rate (GFR) <30 ml/min/1.73 m^2^)
2.6	Patients requiring continuous renal replacement therapy
2.7	Acute liver injury (ALT >500 units/L)
2.8	Patient with acute brain injury
	*After three days he is weaned from Impella and cardiogenic shock is improving. On the fifth day, you lighten sedation to assess neurology for eventual extubation. Regarding your strategy for weaning intravenous midazolam after infusion <5 days:*
2.9	I would cease midazolam with no taper
2.10	I would taper by gradually reducing midazolam dosage over 24–48 h
2.11	I would taper by addition of regular enteral benzodiazepine
2.12	I would taper by addition of as required (PRN) enteral benzodiazepine
2.13	I would only gradually taper following very prolonged infusion (e.g. >5 days)

**Table:** 

	Case 3: Sepsis and acute respiratory distress syndrome
	*A 54-year-old female presents to the ED with shortness of breath and fevers and is diagnosed with community-acquired pneumonia. She has bilateral chest x-ray infiltrates. She progressively deteriorates with severe hypoxaemia and fatigue. She is intubated in your ICU but is immediately difficult to ventilate due to poor compliance and remains hypoxaemic. Regarding sedation in this patient*
3.1	Midazolam as first-line sedation in respiratory failure/acute respiratory distress syndrome
3.2	Midazolam as second-line sedation in patients with ventilator dyssynchrony or failure to achieve lung protective ventilation
3.3	Midazolam as second-line sedation in patients requiring continuous neuromuscular blockade or proning
	*Rate your opinion whether your decision to use midazolam for this patient would change in the presence of one of these conditions:*
3.4	Morbid obesity (body mass index >35)
3.5	Chronic renal insufficiency (GFR <30 ml/min/1.73 m^2^)
3.6	Requiring intermittent dialysis
	*This patient develops severe hypotension, elevated lactate, and multiorgan dysfunction and is diagnosed with severe pneumococcal sepsis. Regarding midazolam use in this setting*
3.7	As first-line sedation in patients requiring 10–20 μg/min (0.2–0.4 μg/kg/min) of noradrenaline to maintain appropriate mean arterial pressure (MAP) targets
3.8	As first-line sedation in patients on 20–30 mcg/min (0.4–0.6 μg/kg/min) of noradrenaline to maintain appropriate MAP targets
3.9	In patients requiring 10–20 mcg/min (0.2–0.4 μg/kg/min) of noradrenaline to maintain appropriate MAP targets while on an alternative first-line sedative agent
3.10	In patients on 20–30 mcg/min (0.4–0.6 μg/kg/min) of noradrenaline to maintain appropriate MAP targets while on an alternative first-line sedative agent
	*High-dose noradrenaline (30 μg/min/0.6 μg/kg/min) and vasopressin (2 units/hr) were initiated to maintain a MAP of*65 mmHg*. A continuous infusion of midazolam was started for haemodynamic stability. Regarding cessation of midazolam in this patient*
3.11	I would routinely cease or taper midazolam once the noradrenaline is below 20 μg/min (0.4 μg/kg/min)
3.12	I would routinely cease or taper midazolam once the noradrenaline is below 10 μg/min (0.2 μg/kg/min)
3.13	I would gradually taper midazolam following shorter duration infusions (e.g., duration <48 hrs)
3.14	I would gradually taper midazolam following longer duration infusions (e.g., duration >48 hrs)

**Table:** 

	Case 4: Traumatic brain injury
	*A 24-year-old male presenting with reduced conscious state following alleged assault was admitted to the ICU following an emergency craniotomy for traumatic intracranial haemorrhage. His initial GCS was 7on the scene. An intracranial pressure (ICP) monitor is sited in theatre, and within four hours of the operation, there are ICP spikes. Regarding sedation for this patient*
4.1	Midazolam as first-line sedation in moderate to severe TBI
4.2	Midazolam as second-line sedation where ICPs are not controlled by propofol and opioid
	*On day 7, there have been no ICP spikes for 48 h and sedation is lightened. Regarding your strategy for weaning intravenous midazolam after prolonged infusions (> 7 days)*
4.3	I would routinely cease midazolam with no taper
4.4	I would taper by gradually reducing midazolam dosage over 24–48 h
4.5	I would taper by addition of regular enteral benzodiazepine
4.6	I would taper by addition of PRN enteral benzodiazepine
4.7	I would gradually taper following very prolonged infusion (e.g., > 5 days)
	*He is waking but tachycardic, tachypnoeic, and agitated. Other sedative agents are added with the view to wean midazolam off. Regarding midazolam cessation in this patient*
4.8	In a patient with multiple lines of sedation (e.g., propofol, clonidine, or dexmedetomidine and enteral agents), I would wean midazolam first
4.9	I am concerned for development of tolerance when using midazolam for >7 days
4.10	I am concerned for addiction and dependence in patients receiving midazolam infusion

**Table:** 

	Case 5: Acute burns and resuscitation
	*A 34-year-old male is brought to the ED after sustaining severe burns in a house fire. He has 65% total body surface area burns involving his face, neck, chest, back, and arms. He is in severe pain and struggling to breath, with signs of airway burns. A decision is made to intubate; however, the airway is severely affected, and after a failed rapid sequence induction, a fibreoptic technique is eventually successful with a size 6.0 endotracheal tube. He is haemodynamically unstable in the ICU, requiring large volume resuscitation and inotropes. Regarding sedation in this patient*
5.1	Midazolam as first-line sedation in acute burns patients due to concerns regarding suitability of propofol in this patient group
5.2	Midazolam as second-line sedation when requiring ongoing volume resuscitation
5.3	Midazolam as second-line sedation when requiring invasive or painful procedures (e.g., dressings or scrubs) that require increased depth of sedation
	*You are concerned about the safety of the endotracheal tube in setting of a difficult airway. A decision is made for increased depth of sedation for tube safety.*
5.4	First-line sedation in patients in whom a Richmond Agitation and Sedation Score (RASS) target of −3 (moderate sedation) to −5 (unresponsive) is required
5.5	First-line sedation in patients requiring a RASS target of −5 (unresponsive)
5.6	Midazolam as second-line sedation in patients whose level of sedation is not adequately controlled on 200 mg/h of propofol
5.7	Midazolam as second-line sedation in patients whose level of sedation is not adequately controlled on 250 mg/h of propofol

**Table:** 

	Case 6: Seizures and out-of-hospital cardiac arrest
	*A female with mild intellectual impairment and epilepsy was brought to the ED following an out-of-hospital cardiac arrest. The arrest was related to hypoxia from aspiration and airway obstruction sustained during a witnessed prolonged generalised seizure lasting over 10 min. Return of spontaneous circulation was following resuscitation of eight minutes, with initial rhythm being pulseless electrical activity. She is brought to your ICU for generalised seizure management and postarrest care, including targeted temperature management. Regarding your sedation choice in this patient*
6.1	Midazolam as first-line sedation in patients who have had a seizure
6.2	Midazolam as first-line sedation in patients in status epilepticus
6.3	Midazolam as second-line sedation if concerns for ongoing seizures despite propofol
6.4	Midazolam as first-line sedation in patients following an out-of-hospital cardiac arrest
6.5	Midazolam as second-line sedation in setting of shivering and/or needing muscle relaxation despite propofol and opioid
	*Rate your opinion whether your decision to use midazolam for this patient would change in the presence of one of the following:*
6.6	Age <50 years
6.7	Age: 50–60 years
6.8	Age: 60–70 years
6.9	Age >70 years
6.10	In patients at risk of confusion (e.g., acquired brain injury and cognitive decline)

CT: computed tomography; ICU: intensive care unit.

## Appendix. Midazolam survey final

Last updated: 7 February 2025

The following cases relate to initiation of midazolam in intubated patients during the first 48 h of admission. Follow-up questions relate to cessation of midazolam at differing time points.

Definitions:

First-line sedation: your initial choice of sedation that would be used as monotherapy (+/- opioid) if sedation targets are achieved
- Second-line sedation: your choice of sedation that you would use in addition to another agent (usually propofol).

Scoring:

Responses are scored from 1: Strongly disagree to 5: Strongly agree.

Consensus will be judged based on 80% agreement for scores 1 and 2 (Strongly disagree, Disagree) or scores 4 and 5 (Strongly agree, Agree).

Use this table as a reference for the frequency you would prescribe midazolam in each situation:

The following cases relate to initiation of midazolam in intubated patients during the first 48 h of admission. Follow-up questions relate to cessation of midazolam at differing time points.
